# Persuasive Features in Web-Based Alcohol and Smoking Interventions: A Systematic Review of the Literature

**DOI:** 10.2196/jmir.1559

**Published:** 2011-07-22

**Authors:** Tuomas Lehto, Harri Oinas-Kukkonen

**Affiliations:** ^1^Oulu Advanced Research on Software and Information SystemsDepartment of Information Processing ScienceUniversity of OuluOuluFinland

**Keywords:** Web-based, online, Internet, alcohol, smoking, intervention, behavior change, persuasive, PSD model, review

## Abstract

**Background:**

In the past decade, the use of technologies to persuade, motivate, and activate individuals’ health behavior change has been a quickly expanding field of research. The use of the Web for delivering interventions has been especially relevant. Current research tends to reveal little about the persuasive features and mechanisms embedded in Web-based interventions targeting health behavior change.

**Objectives:**

The purpose of this systematic review was to extract and analyze persuasive system features in Web-based interventions for substance use by applying the persuasive systems design (PSD) model. In more detail, the main objective was to provide an overview of the persuasive features within current Web-based interventions for substance use.

**Methods:**

We conducted electronic literature searches in various databases to identify randomized controlled trials of Web-based interventions for substance use published January 1, 2004, through December 31, 2009, in English. We extracted and analyzed persuasive system features of the included Web-based interventions using interpretive categorization.

**Results:**

The primary task support components were utilized and reported relatively widely in the reviewed studies. Reduction, self-monitoring, simulation, and personalization seem to be the most used features to support accomplishing user’s primary task. This is an encouraging finding since reduction and self-monitoring can be considered key elements for supporting users to carry out their primary tasks. The utilization of tailoring was at a surprisingly low level. The lack of tailoring may imply that the interventions are targeted for too broad an audience. Leveraging reminders was the most common way to enhance the user-system dialogue. Credibility issues are crucial in website engagement as users will bind with sites they perceive credible and navigate away from those they do not find credible. Based on the textual descriptions of the interventions, we cautiously suggest that most of them were credible. The prevalence of social support in the reviewed interventions was encouraging.

**Conclusions:**

Understanding the persuasive elements of systems supporting behavior change is important. This may help users to engage and keep motivated in their endeavors. Further research is needed to increase our understanding of how and under what conditions specific persuasive features (either in isolation or collectively) lead to positive health outcomes in Web-based health behavior change interventions across diverse health contexts and populations.

## Introduction

In the past decade, the use of technologies to persuade, motivate, and activate individuals’ health behavior change has been a quickly expanding field of research [1-13]. The use of the Web (and related technologies) for delivering interventions has been especially relevant. Automated health behavior interventions have the potential of high reach and low cost [14]. A recent meta-analysis of 75 randomized controlled trials (RCTs) provided support for their effectiveness in changing knowledge, attitudes, and behavior in the health promotion area [15].

There is no consensus on the terminology used to designate the activities conducted over the Internet for mental and physical health purposes. According to Barak et al [16], a Web-based intervention is “a primarily self-guided intervention program that is executed by means of a prescriptive online program operated through a website and used by consumers seeking health- and mental health-related assistance.*”* The intervention in itself is aimed at creating a positive change and/or improving knowledge, awareness, or understanding by providing sound health-related material to the user through an interactive Web-based information system.

Considerable variety exists in terms of types of program content, interactivity, functionality, and level of multimedia of Web-based interventions. In addition, intensity is a major variable; some Web-based interventions are long-term, automated, interactive, tailored, multicomponent programs whereas others are brief online screening instruments with tailored feedback. Finally, participant attrition and exposure rates may vary widely (eg, [18,19]). Technological applications integrating health information with online peer support, decision support, and/or help with behavior change provides an alternative for helping people to achieve better health [20]. However, relatively little is known of designing effective Web-based interventions to support sustained behavior change and improved well-being (eg, [4,21-25]).

The purpose of the present review was to extract and analyze persuasive system features of the included Web-based alcohol and smoking interventions use by applying the PSD model [26]. Suggestions for further research and development are provided for this expanding field of research.

### Persuasive Technology and Health Behavior Change

Research on persuasive technology has been introduced relatively recently [27,28]. Briñol and Petty [29] outline persuasion as follows: “In the typical situation where persuasion is possible, a person or a group of people (ie, *the recipient*) receives an intervention (eg, *a persuasive message*) from another individual or group (ie, *the source*) in a particular setting (ie, *the context*).”

Persuasive systems may be defined as computerized software or information systems designed to reinforce, change, or shape attitudes or behaviors or both without using coercion or deception [26]. Successful persuasion takes place when the target of change (eg, attitudes or beliefs) is modified in the desired direction [29].

Changing people’s behavior is at the heart of health promotion. An individual’s behavior has a significant impact on, for example, cancer and heart disease, which are common causes of premature mortality. The Internet is transforming health care [30] and can be seen as a prime candidate for the application of key behavioral science theories and principles to promote healthier behaviors. There are several advantages in Internet-delivered interventions, for example, reducing cost and increasing convenience for users, reduction of health service costs, reduction of stigma and isolation of users, the need for timely information, and increased user and supplier control of the intervention [31-33]. Internet-based resources, in particular the Web, have many of the characteristics necessary for persuasive communication, and they may provide a channel which integrates the positive attributes of interpersonal and mass communication [34,35]. Web-based systems can give immediate feedback and match the information with the respondent's level of awareness, beliefs, and motivations at that particular time [10]. Additionally, Web-based interventions may overcome isolation of time, mobility, and geography. It has to be noted that Web-based interventions still may be no substitute for face-to-face contact [31,36].

### Persuasive Technology: Designing Systems That Aim at Behavior Change

Examples of persuasive technology can be found quite easily, as there are a variety of websites promoting healthier lifestyles. One of the strongest domains of innovation for persuasive technology in the near future will be preventive health care [37]. On a par with health behavior change, persuasive technology has the potential for significant breakthroughs in many areas of human well-being, such as education and environmental conservation. Nevertheless, the use of persuasive technology in the health arena is still in its infancy. While the field is expanding, it is evident that more research is needed to better determine how the persuasiveness of the systems affects users’ intended behavior.

According to Fogg [38], attempts to create persuasive systems often fail because many projects are too ambitious, being set up for failure. For example, a design team might select a challenging behavior as the target, for example, smoking cessation, but without having ever before created such a persuasive system, the success rate might be low. Zhang [39] stated: "A large number of health information system projects fail. Most of these failures are not due to flawed technology but rather due to the lack of systematic considerations of human and other nontechnology issues in the design and implementation processes." Thus, designing systems that aim at behavior change requires thorough understanding of the problem domain and the underpinning theories and strategies of persuasive systems design. Usually, an interdisciplinary team of professionals is also needed. The main decision points in developing interventions include defining the primary goal of the intervention, defining the target population, and selecting the messages for the intervention [40].

In the present study, the persuasive systems design model (PSD) [26] was applied as the framework for identifying various persuasive techniques that have been incorporated into the Web-based substance use interventions. The PSD model provides a recent and extensive conceptualization of technology-mediated persuasion.

## Methods

### Identification of Studies

We conducted electronic literature searches in five databases (Academic Search Premier [EBSCO Publishing, Herts, England], Cochrane Central Register of Controlled Trials, ISI Web of Knowledge, Medline [Ovid], and Scopus) to identify randomized controlled trials of Web-based interventions for substance use published January 1, 2004, through December 31, 2009, in English.

**Figure 1:**
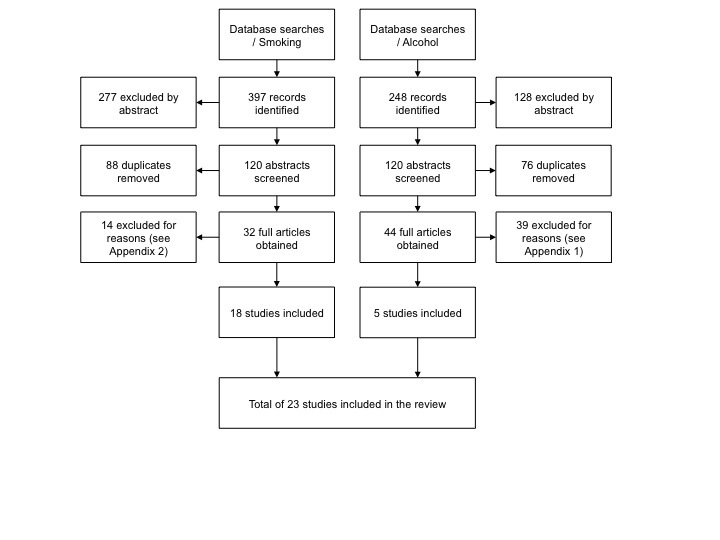
Study identification process

The following terms were used in the database searches: (1) online*, Internet*, web* and (2) intervention, self-help, treatment, trial. To identify alcohol interventions we used the following additional search terms: drink*, alcohol*. For smoking interventions we utilized the terms: smoke*, smoking, cigarette, tobacco, cessation. An asterisk (*) denotes a wildcard. We also screened the bibliographies of relevant articles, including systematic reviews [4,8,10,13,17,41,42] and meta-analyses [5-7,9] to identify potentially relevant studies. The study identification process is depicted in Figure 1.

### Inclusion and Exclusion Criteria

Articles were included if they: (1) focused on Web-based alcohol or smoking (tobacco) interventions, (2) assessed behavioral outcomes or program utilization, (3) were randomized controlled trials or quasi-experimental designs, and (4) were peer-reviewed full research articles.

Follow-up studies that used data from the same cohort of participants were excluded. Both Danaher et al [43] and Severson et al [44] drew data from the same source, but they were included. Brief interventions (see [45, 46]) were excluded because of their rather limited content. Moreover, brief interventions have been commonly reported [47], even to the extent that it was deemed appropriate to put more emphasis on analyzing the less frequently reported, more complex Web-based substance use interventions. According to Moyer [45], a challenge in outlining the research literature on brief interventions is the varying definitions used in different studies. Following Babor’s [46] definition, we included the studies of interventions providing more than three sessions and/or aiming at more than 60 minutes of individual engagement with the program. Furthermore, we excluded articles explicitly using the term *brief intervention* in the title, abstract, or keywords. (See Multimedia Appendices 1 and 2 for excluded articles and reasons these were excluded.)

### Data Abstraction

In total, 23 studies were included in the review and coded. The methodological quality of the included studies was evaluated by applying the CONSORT (Consolidated Standards of Reporting Trials) 2010 checklist [48]. There were some concerns with the quality of 4 studies [44,49-51], but they were still included. Overall, the information presented in the selected articles was thoroughly examined and evaluated. The first author coded all the included articles using a predefined form (devised by the authors) for evaluating persuasive systems. In addition, the abstracted data included various study characteristics (see Table 2). The resulting entries were checked and commented on by the second author. Any disparities were resolved through discussion.

### Persuasive Systems Design (PSD) Model

Information technology always influences people’s attitudes and behavior in one way or another [52,53]. Oinas-Kukkonen and Harjumaa [26,52] have conceptualized a framework for designing and evaluating persuasive systems, known as the persuasive systems design (PSD) model. The PSD model builds on multiple theoretical constructs, such as goal-setting theory [54], elaboration likelihood model [55], and theory of reasoned action/planned behavior [56]. The PSD model is described in full detail elsewhere [26].

Although the PSD model is yet relatively unknown, we consider its use to be justified for this context. In our view, it is the most sophisticated persuasive design and evaluation method available. Many of the principles in the PSD model have been adopted and modified from the seminal work of Fogg [27]. We acknowledge the existence of similar endeavors, such as Ritterband’s behavior model for Internet interventions [22] and Abraham and Michie’s [24] taxonomy of behavior change techniques used in interventions. Despite the similarities and potential overlap, these approaches are quite different.

The PSD model presents a way to analyze, design, and evaluate the persuasion context and related techniques. Persuasion context analysis includes recognizing the intent, the event, and the strategy for persuasion. Dey [57] defines context as follows: “Context is any information that can be used to characterize the situation of an entity. An entity is a person, place, or object that is considered relevant to the interaction between a user and an application, including the user and applications themselves.”

In the PSD model [26], the categories for persuasive system principles are primary task support (supporting the user’s primary task), dialogue support (supporting the interaction between the user and the system), system credibility (the more credible the system is, the more persuasive it is), and social support (the system motivates users by leveraging social influence).

**Figure 2:**
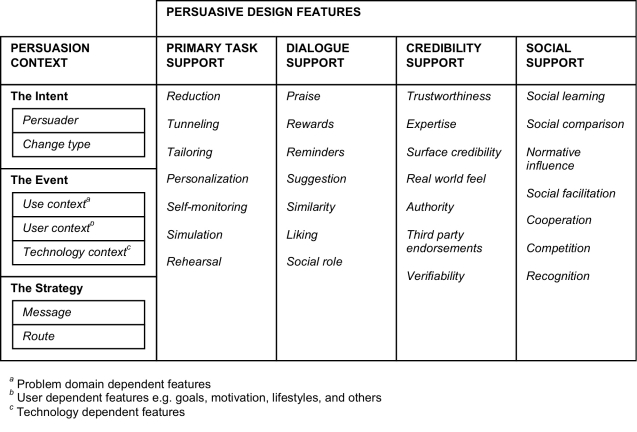
PSD Model (adapted from Oinas-Kukkonen and Harjumaa [26])

The intent includes the persuader and the target behavior change type that the system is to cause in the user. The persuader is the initiator for the development of the system. The event contains the use, user, and technology contexts.

The use context refers to the problem domain dependent features. The use context, in particular problem domain dependent features, is relevant to the persuasion event. The use context can be determined by answering the following questions [58]: Who are the users as a group? What problem-domain dependent features are to be addressed by the design? Who (or what) else is competing for attention in this space?

The user context refers to the individual users characteristics. The user context includes, but is not limited to, users’ (patient, research participant, consumer [22]) characteristics, goals, abilities, and cultural factors. The user context can be clarified by addressing the following questions [58]: What is specific for the users with regard to what they are to be persuaded of? Why is persuasion needed? What constrains their decision?

There is an obvious need for depicting the technology context when describing a Web-based intervention. The technology context refers to the features and requirements of the technological platform and/or application.

The strategy in the PSD model emphasizes two elements, namely the message and the route. The message refers to the form and/or content selected to deliver the intended transformation (eg, behavior or attitude change). The content could be, for instance, statistical data about the health risks of drinking, but the information could be presented to the user in plain text, streaming video, or it could be embedded in a game. The route for persuasion can be direct, indirect, or both. A direct approach provides one or a few solid and convincing arguments, whereas an indirect route is based on a number of facts rather than a single strong argument (compare *central* and *peripheral* routes in ELM). Both routes may be in use simultaneously. A system might represent rational arguments while employing design patterns, which in themselves have been proven persuasive. As an example, an avatar with a specific voice type (angry vs soft spoken or male vs female) to present the message might make the delivery of the content more persuasive for a user; thus, users would be persuaded through the design choices made by the designer.

The persuasive design dimensions and principles are discussed and exemplified below. 

## Results

### Study Characteristics

The characteristics of the included studies are presented in Table 1. Of the 23 articles, 16 targeted smoking, 5, problem drinking, and 2, smokeless tobacco use.

**Table 1:** Characteristics of the included studies

Study Author (Year)	Problem Domain	Primary Objective of the Study	User Context (Number of Participants)	Use and Technology Context (Intervention)	Summary of Findings
An et al (2008) [59]	Smoking	To determine whether an online intervention with college smokers could increase self-reported 30-day abstinence rates at the end of a 2-semester intervention	College smokers at the University of Minnesota (517)	Online college life magazine that provided personalized smoking cessation messages and peer email support (RealU)	The rate of 30-day abstinence at week 30 was higher for the intervention compared with the control group. (41% vs 23%, *P* < .001)
Bersamin et al (2007) [60]	Problem drinking	To assess whether a new online alcohol misuse prevention course is more effective at reducing alcohol use and related consequences among drinkers and nondrinkers	Incoming college freshmen at a northern California public university (622)	Web-based college alcohol education course (College Alc)	Among freshmen who were regular drinkers before college, College Alc reduced the frequency of heavy drinking (d = 0.15), drunkenness (d = 0.09), and negative alcohol-related consequences (d = 0.18). Freshmen who did not report any past 30-day alcohol use before college, College Alc did not experience any beneficial effects.
Brendryen et al (2008) [61]	Smoking	To assess the long-term efficacy of a fully automated digital multimedia smoking cessation intervention	People willing to quit smoking, aged 18 years or older, smoked 10 or more cigarettes daily, and had access to the Internet, email and a cell phone on a daily basis (290)	Fully automated, digital smoking cessation intervention including Web pages, SMS, interactive voice response, emails (Happy Ending)	Participants in the treatment group reported clinically and statistically significantly higher repeated point abstinence rates than control participants. (20% treatment vs 7% control, odds ratio [OR] = 3.43, 95% confidence interval [CI] = 1.60 - 7.34, *P* = .002)
Brendryen et al (2008) [62]	Smoking	To assess the long-term efficacy of a fully automated digital multimedia smoking cessation intervention	People willing to quit smoking, aged 18 years or older, smoked 10 or more cigarettes daily and had access to the Internet, email, and a cell phone on a daily basis (396)	Fully automated, digital smoking cessation intervention including Web pages, SMS, interactive voice response, emails (Happy Ending)	Participants in the treatment group reported clinically and statistically significantly higher repeated point abstinence rates than control participants. (22.3% treatment vs 13.1% control; OR = 1.91, 95% CI 1.12 - 3.26, *P* = .02)
Buller et al (2008) [63]	Smoking	To reduce smoking by children in grades 6 through 9 by convincing those who had not smoked not to start and persuading those who had already tried smoking to stop	Sixth to ninth graders from Australia and the United States (2077)	Tailored, Web-delivered smoking prevention program for adolescents (Consider This)	No statistically significant differences between groups were found
Danaher et al (2006) [43]	Smokeless tobacco use	To define participant exposure measures to a Web-based program for smokeless tobacco cessation	Recruited smokeless tobacco users (2523)	Web-based smokeless tobacco cessation intervention (ChewFree.com, enhanced)	Participants in the enhanced condition made more visits and spent more time accessing their assigned website than did participants assigned to the basic condition website.
Escoffery et al (2004) [51]	Smoking	To develop and conduct a process evaluation of a Web-based smoking cessation intervention for college smokers	College smokers (70)	Web-based smoking cessation program for college smokers (Kick It!)	No statistically significant differences between groups

Etter (2005) [64]	Smoking	To compare the efficacy of two Internet-based, computer-tailored smoking cessation programs	College smokers (11,969)	Web-based, computer-tailored smoking cessation (Stop-tabac.ch)	Statistically significant differences in quit rates in smokers in the contemplation stage favoring the original program. (OR = 1.54, 95% CI = 1.18 - 2.02, *P* = .002)
Finfgeld-Connett and Madsen (2008) [49]	Problem drinking	To evaluate the effectiveness of a Web-based, self-guided treatment program for women with problem drinking habits who live in rural areas of Missouri	Adult women with problem drinking habits living in Missouri counties (44)	Web-based, self-guided treatment program for problem drinking (intervention name not reported)	No statistically significant results
Hester et al (2009) [65]	Drinking moderation	To evaluate the effectiveness of a Web-based moderation training	Heavy drinkers (84) who responded to a newspaper recruitment ad (Albuquerque, New Mexico) Alcohol Use Disorders Identification Test (AUDIT) scores >7; Drinking >10 standard drinks per week; not currently abstaining; interest in moderating their consumption; aged 21 or older; Internet access at home	Internet-based program and use of the online resources of Moderation Management (MM)	At 3-month follow-up both groups significantly reduced their drinking. Both groups also significantly reduced their alcohol-related problems. Relative to the control, the experimental group had better outcomes on percent days abstinent.
Japuntich et al (2006) [50]	Smoking	To evaluate the impact of the program in an efficacy evaluation context	Smokers (at least 18 years old) motivated to quit smoking, 134 participants were recruited in a research center in Milwaukee, Wisconsin; 150 participated in a research center in Madison, Wisconsin (284)	Web-based smoking cessation and relapse prevention intervention (CHESS SCRP)	No statistically significant differences between groups
Matano et al (2007) [66]	Problem drinking	To pilot test an interactive Web-based intervention for reducing alcohol consumption	Employees of a work site in the Silicon Valley region of California, categorized as low or moderate risk for alcohol problems (145)	Interactive Web-based intervention for reducing alcohol consumption (CopingMatters)	The sample size was inadequate for evaluating treatment effects on drinking [66].
McKay et al (2008) [67]	Smoking	To describe the 6-month follow-up results of an RCT where participants were randomly assigned to either a Web-based smoking cessation program or a Web-based exercise enhancement program	Smokers at least 18 years of age interested in quitting within the next 30 days, willing to engage in moderate physical activity, access to the Internet (2318)	Web-based tailored smoking cessation (Quit Smoking Network; QSN)	No between-condition differences in smoking abstinence were found at 3- and 6-month follow-up assessments.
Muñoz et al (2006) [68]	Smoking	To compare a standard smoking cessation intervention to the same guide plus a mood management intervention	English- or Spanish-speaking smokers; ≥ 18 years old, smoking ≥ 5 cigarettes/day, using email at least once weekly and intending to quit in the next month; recruited from general population in USA (568)	Web-based intervention providing standard cessation information, tailored advice; individually timed educational messages (ITEMs); online mood management (MM) course (Guía)	ITEMs increased the effectiveness of the Guía. However, MM reduced quit rates, at times significantly so.

Muñoz et al (2009) [69]	Smoking	To examine abstinence rates of an Internet smoking cessation intervention and whether providing additional elements to a static Internet stop-smoking guide increases them	Spanish- and English-speaking participants (worldwide) were recruited using online campaigns; ≥ 18 years old, smoking ≥ 5 cigarettes/day, using email at least once weekly and intending to quit in the next month (1000)	Condition 1 was the “Guía Para Dejar de Fumar,” a static National Cancer Institute evidence-based stop smoking guide; Condition 2 consisted of Condition 1 plus ITEMs; Condition 3 consisted of Condition 2 plus MM; and Condition 4 consisted of Condition 3 plus a “virtual group” (an asynchronous bulletin board)	No significant differences among the four conditions were found.
Patten et al (2006) [70]	Smoking	To test the efficacy of a home-based, Internet-delivered treatment for adolescent smoking cessation	Adolescent smokers aged 11-18 years (139)	Web-based smoking cessation intervention tailored to adolescents (Stomp Out Smokes; SOS)	No statistically significant differences between groups were found.
Riper et al (2007) [71]	Problem drinking	To determine the effectiveness of a self-help intervention for adult problem drinkers	Adult Dutch problem drinkers (261)	Web-based self-help intervention (Drink Less)	At follow-up, 17.2% of the intervention group participants had reduced their drinking within the guideline norms; in the control group this was 5.4% (OR = 3.66, 95% CI = 1.3-10.8, *P* = .006, number needed to treat [NNT] = 8.5). The intervention subjects decreased their mean weekly alcohol consumption significantly more than control subjects, with a difference of 12.0 standardized units (95% CI 5.9 - 18.1, *P* < .001, standardized mean difference 0.40).
Severson et al (2008) [44]	Smokeless tobacco use	To test the impact of an interactive, tailored Web-based intervention versus a more linear, text-based website	Recruited smokeless tobacco users (2523)	Interactive, tailored Web-based intervention (ChewFree.com, enhanced)	Participants in the enhanced condition quit at significantly higher rates (vs basic condition). Abstinence was 40.6% in the enhanced condition vs 21.2% in the basic condition (*P* < .001). Using intent-to-treat analysis, quit rates were 12.6% vs 7.9% (*P* < .001)
Stoddard et al (2008) [72]	Smoking	To determine the use and satisfaction with two versions of a smoking cessation website, one of which included an asynchronous bulletin board	Adult federal employees or contractors to the federal government who responded to an email and indicated a willingness to quit smoking in 30 days (1375)	Smoking cessation website (Smokefree.gov, added bulletin board)	No statistically significant differences between groups were found.(Time spent on the website was significantly longer for the intervention subjects than for the control subjects.)

Strecher et al (2005) [73]	Smoking	To assess the efficacy of Web-based tailored behavioral smoking cessation program among nicotine patch users	Smokers in the United Kingdom and Republic of Ireland who purchased a certain patch and connected to a website (3971)	Web-based tailored behavioral smoking cessation (CQ Plan)	Continuous abstinence rates at 6 weeks were 29.0% in the tailored condition vs 23.9% in the nontailored condition (OR = 1.30, *P* < .001), at 12 weeks continuous abstinence rates were 22.8% versus 18.1%, respectively (OR = 1.34, *P* < .001) (Satisfaction with the program was significantly higher in the tailored than in the nontailored condition)
Strecher et al (2008) [74]	Smoking	To determine (1) whether engagement in a Web-based smoking cessation intervention predicts 6-month abstinence, (2) whether certain groups are more likely to have lower engagement, and (3) whether particular program components influence engagement	Smokers, participants from two large health maintenance organizations (1866)	Web-based program for smoking cessation and relapse prevention (intervention name not reported)	The total number of Web sections opened was related to subsequent smoking cessation. More personalized source and high-depth tailored self-efficacy components were related to a greater number of Web sections opened.
Swartz et al (2006) [75]	Smoking	To test the short-term efficacy of an automated behavioral intervention for smoking cessation delivered via a website	18 years or older, smoking cigarettes on a daily basis, considering quitting smoking in the next 30 days, and being able to access the website. (351)	Video-based website (1-2-3-Smokefree)	At 90 days, the cessation rate was 24.1% for treatment group versus 8.2% for the control group (*P* = .002). Using an intent-to-treat model, 12.3% of the treatment group were abstinent versus 5.0% in the control group (*P* = .015)
Woodruff et al (2007) [76]	Smoking	To test an innovative approach to smoking cessation that might be particularly attractive to adolescent smokers	Adolescent smokers in high school (136)	Web-based counseling program, virtual world chat room for adolescent smoking cessation (Breathing Room)	At the immediate postintervention assessment, intervention group participants were significantly more likely to report that they had abstained from smoking during the past week (*P* < .01), smoked fewer days in the past week (*P* < .001), smoked fewer cigarettes in the past week (*P* < .01), and considered themselves former smokers (*P* < .05). At a 1-year follow-up assessment, only the number of times quit was statistically significant (*P* < .05).

### Persuasion Context: The Intent

#### Persuader

All of the articles stated a primary objective of the study (see Table 2), thus revealing a motive to persuade the users of the system. Most commonly, the Web-based interventions seemed to have been established by relatively small teams of people with varying expertise and background.

#### Change Type

In 8 of the articles, the intended change type was explicated. There were examples of reporting the intended change type in a simple and clear manner. For example,

“The self-help program proceeds in four successive stages: (1) preparing for action; (2) goal setting; (3) behavioral change; and (4) maintenance of gains and relapse prevention” [71]; “Participants were reassessed after 90 days for their alcohol consumption to assess changes in their drinking behavior” [66]; and “The QSN condition provided smoking cessation information and behavior change strategies while the Active Lives condition provided participants with physical activity recommendations and goal setting” [67].

### Persuasion Context: The Event

#### Use Context

Our findings confirm the claim put forward by Griffiths and colleagues [31], who stated as a result of their systematic review that a number of studies gave no reason for using the Internet as the mode of delivery other than stating that the software application exists and needs to be evaluated. None of the articles reported the use context, for example, user groups, problem-domain dependent features, and attention drawers in detail. A high abstraction level in system descriptions makes it difficult to grasp what kind of interaction, and under what circumstances, really takes place through the system and to what extent the potential outcome really is due to the intervention.

#### User Context

The user context refers to characteristics of the individual user. All articles reported the user context to some extent. The authors of published articles may possess detailed information about users and their individual characteristics, but for some reason it does not seem to be clearly reflected in the dissemination.

#### Technology Context

The interventions included in the review were Web-based, and all of them aimed at persuading the users in some way. The majority of the research articles (14 out of 23) presented the technology context concisely. Screenshots are worthwhile, even though they do not reveal the backbone of the system, the information architecture, or the flow between parts of the system and its content. Escoffery et al [51] reported a brief flowchart of the development and evaluation process of the intervention, and Swartz et al [75] presented an overview flowchart of the intervention program. In our opinion, these types of charts are beneficial in helping readers to understand what actually takes place (and when) through the interventions.

### Persuasion Context: The Strategy

#### Route

All of the reviewed studies seemed to rely on indirect routes for persuasion. However, due to the limited descriptions of the route utilized, it was not possible to confirm this fully. Of the studies reviewed, 14 revealed the underlying theories or methods behind the intervention to some extent. The most common theories were social cognitive theory (in 5 studies) and stages of change (in 4 studies), while in 5 of the studies the application of cognitive-behavioral methods was reported.

#### Message

One of the key decision points in developing interventions is the selection of messages for the intervention [40]. According to Ritterband et al [22], the message focuses on the source and style of the content, and it provides important information about who created the content and how it was presented for the users. These are hypothesized to impact user engagement and other mechanisms of change, including the acquisition of knowledge and motivation. In the PSD model, *the source* is called *the persuader*. All of the reviewed articles described the message at least partially.

### Persuasive Features: Primary Task Support

The functionalities in the primary task category support the carrying out of the user’s primary task. Persuasion techniques in this category [26] include *reduction, tunneling, tailoring, personalization, self-monitoring, simulation, and rehearsal* (compare Fogg [27]). These were reported relatively well in the reviewed studies.

#### Reduction

All of the reviewed articles described functionality countable as reduction, that is, the system reduces complex behavior into simple tasks helping users to perform the target behavior. This is important because a system that guides users through a process or experience provides opportunities to persuade along the way. For instance, in spite of the alcohol treatment program described in Finfgeld-Connett and Madsen [49], being complex (consisting of 8 reference modules and 15 decision-making modules) its use seemed to have been made easy for the end-users. For another instance, Swartz et al [75] described the modular structure of the intervention as “benefits of stopping smoking, overcoming common barriers to cessation, strategies for avoiding situations that prompt cravings, strategies for dealing with cravings, and setting a quit date.”

#### Self-monitoring

A system keeping track of the user’s performance or status supports achieving one’s goals. Not surprisingly, this type of self-monitoring functionality was found in all of the included articles. For example, the system described in Escoffery et al [51] allowed participants to review their smoking by sending immediate feedback forms and copies of the personalized assessments to their email accounts. An et al [59] reported that participants received a weekly email invitation to visit the study website to report on health and lifestyle habits for the prior week, whereas Finfgeld-Connett and Madsen [49] described decision-making modules that included daily alcohol monitoring. Brendryen et al [61] stated that a major focus in their application was to ensure that participants comprehended that self-awareness, self-monitoring, active participation, and engagement are essential elements for reaching personal goals.

#### Simulation

Enabling users to observe the link between the cause and its effect is regarded as simulation. Simulations that educate users about certain topics can leave a lasting impact that transfers to the real world [77]. In all, 14 interventions featured at least some sort of simulation. For instance, Stoddard et al [72] described an interactive smoker’s risk tool simulating changes in the risk of death due to smoking based on the smoker’s history and time of quitting. In the Woodruff and colleagues [76] study, a virtual pathology lab showed pictures of damaged organs and premature aging, providing a scenario for discussing the short- and long-term health effects of smoking.

#### Personalization

A persuasive system may offer personalized content and services for its users. In order for the content to be personalized, the user has to disclose some personal information about herself, for example, through registration or by creating a personal profile [78]. The quality of Web personalization depends on how well the content generated by the personalization agent matches the preferences of the user in a particular domain [79]. Features of personalization were described in 13 articles. An et al [59] reported that email message content was personalized by peer coaches using information provided by participants during their weekly visits to the website. The enhanced website in Severson et al [44] included a guided, interactive program (“Personal Quitting Assistant”) to help each user create an individual plan for quitting smokeless tobacco and preventing relapse. The intervention in Swartz et al [75] included creating a personalized quit plan calendar with individualized tips.

#### Tailoring 

According to Rimer and Kreuter [80], studies related to tailoring should explore how and under what circumstances tailoring works and how its effects may be optimized. Tailoring the content to meet the potential needs, interests, personality, usage context, and/or other factors relevant to a user group is likely to increase the persuasiveness of the system. In all, 8 articles described tailoring functionality embedded in the actual interventions. For example, Strecher et al [73] reported that information collected in the enrollment questionnaire of their application was used to tailor the Web-based intervention materials. McKay et al [67] described tailoring portions of the program content to match each participant’s smoking status in their application. The intervention in Swartz et al [75] consisted of 13 separate versions or strands, including 12 demographically targeted versions and 1 multicultural version, each with the same basic structure and content. The tailored versions were based on user’s sex, age, and ethnicity.

#### Tunneling

Tunneling may enhance the change process since the user is led through a predetermined sequence of steps and receives the most appropriate content, particularly at a proper time [43]. A total of 4 articles described tunneling functionality. Buller et al [63] reported that program progression was controlled in their case by teachers. The *current* and *previous* modules were available to students, but students could not progress forward into subsequent modules until the teacher revealed the password to do so. In McKay et al [67], the intervention condition utilized hybrid information architecture (see [81]) where first-time users were tunneled through a series of tailored pages in order to introduce them to the key concepts and strategies of a behavioral program for quitting smoking. Severson et al [44] reported that in their “Planning to Quit” module users progressed linearly through content addressing readiness to quit, reasons for quitting, and level of dependence. In Hester et al [65], users were able to go through the program’s modules sequentially, or they could choose the most relevant modules for them.

#### Rehearsal

Rehearsing a behavior can enable people to change their attitudes or behavior in the real world. Only 1 article reported this type of functionality; virtual locations (in the “Breathing Room” virtual world) such as a teen dance club and a fast food restaurant provided settings to discuss social influence to smoke and relapse, and an amphitheater was available for additional virtual meetings and discussions [76].

### Persuasive Features: Dialogue Support

Dialogue support defines the key principles in keeping the user active and motivated in using the system and helping the user to reach the intended behavior. The principles in the dialogue support category are *praise, rewards, reminders, suggestion, similarity, liking,* and *social role*. Surprisingly, dialogue support was an underreported area in the reviewed papers.

#### Reminders

A persuasive system should remind users of their target behavior during the use of the intervention. A recent systematic review showed that the use of periodic prompts could be effective in behavior change interventions [82]. Of the reviewed studies, 10 utilized these kinds of reminders. In Stoddard et al [72], all participants received email reminders. During the first 2 weeks of their study on smoking cessation, participants received four email reminders unless they had previously expressed their wish to discontinue their participation. Each email included advice on quitting, a brief message encouraging use of the intervention, as well as the time frame of the future follow-up assessments. In Brendryen et al [61], if users did not log on to the program or answer the log-off call, they received a reminder call and up to two reminder text messages. The reminders were fully automated. Japuntich et al [50] described nonautomated reminders; if participants went a week without logging onto the system, the staff telephoned them and reminded them to log in.

#### Social Role

A system adopting a social role (eg, doctor or teacher) may be more persuasive. None of the reported interventions seemed to utilize social role per se. However, 9 of the articles described ask-an-expert service, which is related to social role (but also falls under expertise and authority in credibility support).

#### Suggestion

A system should provide the user with fitting suggestions at proper moments during the system use. This kind of suggestion was featured in 4 articles. In Japuntich et al [50], the program suggested different articles or other services to the user based on his or her responses to the *check-* *in* (eg, smokers reporting depression were encouraged to use the cognitive behavioral therapy service). At the start of each session, the program in McKay et al [67] displayed online prompts recommending the review of content that a participant had not yet explored. In Stoddard et al [72], the program provided *Did you know?* messages containing appropriate links and information. The content of the program in Strecher [73] included suggestions for coping with nicotine replacement therapy and encouraging compliance.

#### Similarity

Individuals are more readily persuaded through systems resembling themselves in some meaningful way. For example, a system aimed at teenagers should employ youthful phrases and imagery. This principle is known as similarity (compare *social identity cues* [78]), and it was found in 4 of the articles. In Escoffery et al [51], college smokers provided ideas for components to be incorporated into the Web-based intervention, for example, graphical presentations of information, providing only small amounts of text, sharing of stories/situations, quizzes, and other interactive features. These were then taken into account in the development of the program content. Similarity was utilized in Bersamin et al [60] as four of the five units in the program for college students (“College Alc”) included a streaming video clip depicting college students in an alcohol-related context. In addition, it utilized student-generated harm-prevention plans.

#### Liking

An attractive system is likely to be more persuasive. This principle is known as *liking,* and it was addressed in 3 articles. Woodruff et al [76] stated that the primary goal of their application was to test an innovative approach for smoking cessation that might be particularly appealing to adolescent smokers. In Buller et al [63], audio narration, graphics, animation, sound effects, and music were utilized to create a rich multimedia environment to stimulate user engagement. In Escoffery et al [51], as a part of the formative research before the development of the program, college smokers were asked about their Internet usage and features that they liked to learn about potential elements to be added into the Web application.

#### Rewards

The system should reward the user for achieving self-set goals, for example [54]. None of the articles reported this type of automated functionality. An example implementation could be, for instance, that the user would be rewarded with a virtual trophy upon completion of a certain task. However, Severson et al [44] reported that their *Staying Quit* module provided tailored information and behavioral strategies on eight major topics including *Reward Yourself*.

#### Praise

A system could praise users via words, images, symbols, or sounds based on their behaviors. By offering praise, a system can make users more open to persuasion. Quite surprisingly, this technique was not featured in any of the reviewed studies.

### Persuasive Features: Credibility Support

Credibility is a persuasive element (eg, [78,83,84]). Harris et al [85] suggested that perhaps even seemingly superficial design elements of a website can influence responses to health-risk information. In their study, credibility cues affected the engagement with the site and influenced subsequent health behavior and cognition. According to Briñol and Petty [29], confidence in one’s thoughts is likely to be undermined if the received message is not credible.

The PSD model describes seven principles for supporting system credibility: *trustworthiness, expertise, surface credibility, real world feel, authority, third party endorsements, and verifiability*. The analysis of these is more a continuum than a dichotomy. For this reason, explicit numbers and percentages are not given here. However, based on the textual descriptions of the interventions, we suggest that most of them incorporated—to a notable degree—expertise (system provides information demonstrating knowledge, experience, and competence), verifiability (system provides means to verify the accuracy of site content via outside sources), authority (system quotes an authority, such as a statement or norms by an authoritative health institute), and trustworthiness (system provides truthful, fair, and unbiased information). Trustworthiness is crucial in website engagement as users will engage with sites they perceive trustworthy and navigate away from those they mistrust [78].

Naturally, evaluating perceived credibility is more or less subjective. People make initial assessments of the system credibility based on a firsthand inspection. This principle is called *surface credibility*. A persuasive system should provide information of (and means to communicate with) the organization and/or actual people behind its content and services. This feature is called *real-world feel*. To fully evaluate both surface credibility and real-world feel would require a hands-on approach on the actual implemented interventions.

A persuasive system could also provide endorsements from respected and renowned sources, for example, a recommendation by an authoritative health organization, an award for excellence in usability, or a privacy seal to ensure confidentiality. However, none of the articles reported utilizing third party endorsements.

### Persuasive Features: Social Support

According to Uchino [86], social support may refer to the aspects of the social network (groups or familial ties), specific behaviors (eg, emotional or informational support), or our perceived availability of support resources that may be shaped early in life. In the PSD model, the social support category describes how to design the system so that it motivates users by leveraging social influence. The model operates with the following persuasion techniques: *social learning, social comparison, normative influence, social facilitation, cooperation, compensation,* and *recognition*.

The social learning principle means that individuals may be more motivated to perform a target behavior if they can observe others performing the behavior while using the system. A closely related principle is social comparison: users will be more motivated to perform the target behavior if they can compare their performance with the performance of others. Users are also more likely to perform target behavior if they are able to observe others performing the behavior or are being observed by others. This principle is called *social facilitation*.

The most common means to providing social support were asynchronous peer discussion forums and synchronous chat rooms. A variety of other online social support features were also utilized. For a full description of studies regarding social learning, social comparison social facilitation, and normative influence, see Table 2.

**Table 2:** Social support in the Web-based interventions

Study Author (Year)	Social Learning, Comparison and/or Facilitation	Normative Influence	Other Support
An et al (2008) [59]	No	Not reported	Email exchange with peer coach
Bersamin et al (2007) [60]	Discussion forum	Streaming video clips, College Alcohol Use unit	Not reported
Brendyen et al (2008) [61,62] (Note: 2 articles are combined here as they both study the Happy Ending system)	No	Not reported	Pre-recorded audio messages for relapse prevention, Interactive Voice Response-based craving helpline
Buller et al (2008) [63]	No	Prevention content focused on social influence and aimed, for example, to correct inexact perceptions of tobacco use norms	Not reported
Danaher et al (2006) [43] Severson et al (2008) [44] (Note: these articles are combined because they study the same website with the same participants)	Peer-to-peer forum	Testimonial videos	Ask-an-expert forum
Escoffery et al (2004) [51]	Stage-matched discussion forums	Shared personal stories area	Not reported
Etter (2005) [64]	Discussion forums, chat rooms	Personal stories written by current and former smokers	Not reported
Finfgeld-Connett and Madsen (2008) [49]	Asynchronous bulletin board, synchronous chat featured	Not reported	Private messaging to the researcher and other users
Hester et al (2009) [65]	Online mutual-help support community	Not reported	Online and face-to-face meetings
Japuntich et al (2006) [50]	Discussion group, chat room (trained counselor available)	The smoking-related topics included facts about smokers, smoking, and cigarette companies	Ask-an-expert service
Matano et al (2007) [66]	No	Individualized feedback with normative data	Not reported
McKay et al (2008) [67]	Peer-to-peer forum	Not reported	Ask-an-expert forum
Muñoz et al (2006, 2009) [68,69]	Asynchronous bulletin board	Not reported	Mood management online course
Patten et al (2006) [70]	Discussion support group	Videos of personal stories	Private email service with an expert
Riper et al (2008) [71]	Moderated peer-to-peer discussion forum	Streaming video (quitting information and testimonials)	Not reported
Stoddard et al (2008) [72]	Asynchronous bulletin board	Not reported	Ask-an-expert forum
Strecher et al (2005) [73]	No	Not reported	Behavioral support email messages
Strecher et al (2008) [74]	No	A section with a narrative success story	Not reported
Swartz et al (2006) [75]	No	Personalized video segments	Not reported
Woodruff et al (2007) [76]	Virtual world chat room, virtual locations	Topics covered, for example, peer influence; virtual locations in “Breathing Room” virtual world	Smokers interacted with each other as well as with the counselor

A system can apply normative influence, in other words, positive peer pressure to enhance the likelihood that an individual will adopt a target behavior. Buller et al [63] and Matano et al [66] reported that users were working individually with the program to create a sense of privacy so they would unfold their smoking intentions.

In addition to the aforementioned ways for providing social support, a system can persuade users to adopt a target attitude or behavior by leveraging human beings’ natural drive to compete and cooperate. Competition was reported in 2 and cooperation in 1 of the analyzed papers. In An et al [59], the intervention site actively promoted a “Quit and Win” contest and included links to the online sign-up for this contest. An example of cooperation was found in Woodruff et al [76], as the students were working as a group in the virtual environment.

By offering public recognition to an individual or a group, a system can increase the likelihood that a person and/or group will adopt a target behavior. Only 1 of the articles presented functionality countable as recognition. In Woodruff et al [76] a message, “Guero's [a person’s name] been quit for a month!” was presented on billboards throughout the virtual world. 

## Discussion

### Findings

Of the 23 articles included in the review, 20 primarily measured health behavior outcomes. Of these articles, 12 reported statistically significant differences between groups. Overall, 3 articles assessed program utilization, and 2 of them reported positive findings (see Table 1).

The primary task support components were reported relatively widely in the reviewed studies. Reduction, self-monitoring, simulation, and personalization seem to be the most used ways to support accomplishing a user’s primary task. This is an encouraging finding because reduction and self-monitoring can be considered as the key elements of primary task support. The utilization of tailoring was surprisingly low. The lack of tailoring may imply that the interventions are targeted for too broad an audience. It would be reasonable to assume that different approaches were needed for different kinds of user groups. Elements of dialogue support were mostly underutilized in the interventions. Leveraging reminders [82] was the most common way to enhance the user-system dialogue.

Credibility issues are crucial in website engagement as users will engage with sites they perceive credible and navigate away from those they do not find credible. Based on the textual descriptions of the interventions, we cautiously suggest that most of them were relatively credible. Nevertheless, it seems to be, as Danaher and Seeley [19] eloquently put it, that “credibility is in the eye of the beholder.”

The prevalence of social support in the reviewed interventions was encouraging (see Table 2). Social support provided by the systems is often based on peer support. According to Eysenbach [36], there is not yet very strong evidence for what type of peer-based social support the systems really should provide. In their systematic review, Shahab and McEwen [17] argued that while chat forums could potentially aid in stopping smoking through the providing extra social support, it still seems to be that entirely automated interventions are even more effective. Bennett and Glasgow [14] claimed that there are no examples of trial designs that would enable a systematic investigation of the potential benefits of social networking. In this review, we were not able to confirm the effect of social support components on health behavior outcomes. This is mainly due to the fact that only 1 of the included articles [72] explicitly studied the effect of adding a social component (virtual community) within the intervention. Furthermore, many research and design issues still need to be resolved about the type of online support, for example, whether it should be expert-led versus user-driven, moderated versus unmoderated, synchronous versus asynchronous, or open access versus restricted access, among other issues.

### Applying the Persuasive Systems Design Model

Evaluating the effectiveness of specific persuasive features within Web-based interventions is difficult since the features are not usually explicitly tested. According to Kypri and Lee [87], the descriptions and analyses of how interventions are developed are often absent from scientific literature due to space constraints. However, we think that the presentation of detailed information about the theoretical basis, functionality, content, and structure of a Web-based intervention helps to interpret the results and conduct evaluations as on a more finely grained level. Ahern [88] points out that randomization to experimental groups or conditions remains the gold standard for evaluating intervention efficacy but may not provide the most relevant information for dissemination. Furthermore, methodological challenges and latent scientific foundations in researching Internet-based interventions are acknowledged by many researchers (eg, [19]). Glasgow [89] stated that the diversity in content area, disciplines involved, and publication outlets is the reason that the consistency in how Internet-based solutions have been conceptualized, reported, and evaluated has been low. Finally, the brevity of intervention descriptions makes it more difficult to draw generalizable results (see [8,13]).

Due to these issues, the application of the PSD model turned out to be relatively laborious. In the present review, we relied on textual descriptions of the interventions, thus being able to provide only a limited synthesis. Regardless of its wide coverage, the PSD model is not an exhaustive list of persuasive features, and also some of the features are overlapping (eg, social learning/comparison/facilitation and liking/similarity) and thus rather difficult to analyze. New persuasion techniques may also be identified in the future. The PSD model has been built in such a manner that it may evolve, but even as it stands now, it is an important asset for any health behavior change system developer.

### Limitations and Strengths of the Review

Analyzing persuasive design is a challenging task. When conducting an analysis such as described in the present review, potential bias lies in the interpretation of the articles. Nevertheless, in extracting and categorizing persuasive features, we rigorously observed if the authors clearly stated the described variables. Obviously, the articles did not necessarily follow the very same terminology as found in the PSD Model. Thus, the analysis was based on interpretive categorization.

This systematic review focused on randomized controlled trials, thus excluding potentially meritorious studies. (Quasi-experimental studies were not found in the search process.) A meta-analysis was not conducted due to the heterogeneity of the studies. Overall, there are already several reviews on Web-based (or similar) interventions. To our knowledge, the present review is the first systematic review to address persuasive system features in Web-based interventions for substance use.

### Conclusion

In this review we examined the persuasive system features of the included Web-based interventions. We think that this type of novel approach is useful for current and future research for recognizing what kind of tactics in present systems have been utilized to motivate people in achieving better health.

However, at this point, linking specific persuasive features to outcomes is difficult, relying only on brief textual descriptions of the interventions. Also, it is not possible to determine the (perceived) credibility of a Web-based intervention based on reading an article. We acknowledge that many studies have examined, for example, the role of tailoring in health behavior change interventions [10,11], but the *persuasiveness* of a particular component/application/system is a more complex issue and has yet to be tackled in future endeavors.

We are not implying that the mere presence of persuasive features is enough. The development of Web-based and other similar interventions is a highly elaborate and a multifaceted issue. Still, it is relevant to consider the technological aspects since the Web and related technologies are being used as a delivery channel. Atienza and colleagues [90] pertinently remarked that “health information technology does not occur in a vacuum, but rather technologies exist within social systems.” In order for widespread adoption, dissemination, and extended use of technology-enabled health behavior change interventions to take place, it is necessary to investigate not only how the interventions affect individuals, but also how individuals interact with technology and each other [90]. Further research is also warranted to increase our understanding of how and under what circumstances specific persuasive features (either in isolation or collectively) lead to positive health outcomes in Web-based health behavior change interventions across diverse contexts and populations.

## Multimedia Appendix 1

Excluded articles (N=39): Alcohol

## Multimedia Appendix 2

Excluded articles (N=14): Smoking

## Abbreviations

AUDIT: Alcohol Use Disorders Identification Test
CI: confidence interval
CONSORT: Consolidated Standards of Reporting Trials
ITEM: individually timed educational message
MM: mood management
NNT: number needed to treat
OR: odds ratio
PSD: persuasive system design
RCT: randomized controlled trial
SoSE: Graduate School on Software Systems and Engineering
TEKES: Finnish Funding Agency for Technology and Innovation
