# Supplementary material for: Persuasive Features in Web-Based Alcohol and Smoking Interventions: A Systematic Review of the Literature
Source: J Med Internet Res. 2011 Jul 22;13(3):e46. doi: 10.2196/jmir.1559 (PMC3222186; doi:10.2196/jmir.1559)
Supplement: Supplementary file 1 [file jmir_v13i3e46_app1.pdf]

Tuomas Lehto and Harri Oinas-Kukkonen:
Persuasive Features in Web-Based Alcohol and Smoking Interventions – A Systematic Review of the Literature

MULTIMEDIA APPENDIX 1. EXCLUDED ARTICLES (N=39) / ALCOHOL

SUMMARY OF REASONS (N):

- Brief intervention (30)
- Face-to-face components or interaction with participants (2)
- Non-RCT (2)
- Review (1)
- Protocol (1)
- Summary of proceedings (1)
- No major web component (1)
- Secondary analysis (1)

BRIEF INTERVENTION (E.G. SCREENING AND FEEDBACK) (N=30)

1. Bewick BM, Trusler K, Barkham M, Hill AJ, Cahill J & Mulhern B. (2008). The effectiveness of web-based interventions designed to decrease alcohol consumption--a systematic review. Preventive Medicine, 47(1), 17-26.

2. Bewick BM, Trusler K, Mulhern B, Barkham M & Hill AJ. (2008). The feasibility and effectiveness of a web-based personalised feedback and social norms alcohol intervention in UK university students: a randomised control trial. Addictive Behaviors, 33(9), 1192-8.

3. Blankers M, Koeter M & Schippers GM. (2009). Evaluating real-time internet therapy and online self-help for problematic alcohol consumers: a three-arm RCT protocol. BMC Public Health, 9, 16.

4. Chiauzzi E, Green TC, Lord S, Thum C & Goldstein M. (2005). My student body: a high-risk drinking prevention web site for college students. Journal of American College Health, 53(6), 263-74.

5. Croom K, Lewis D, Marchell T, Lesser ML, Reyna VF, Kubicki-Bedford L, et al. (2009). Impact of an online alcohol education course on behavior and harm for incoming first-year college students: short-term evaluation of a randomized trial. Journal of American College Health, 57(4), 445-54.

6. Cunningham JA, Humphreys K, Kypri K & van Mierlo T. (2006). Formative evaluation and three-month follow-up of an online personalized assessment feedback intervention for problem drinkers. Journal of Medical Internet Research, 8(2), e5.

7. Cunningham JA, Wild TC, Cordingley J, van Mierlo T & Humphreys K. (2009). A randomized controlled trial of an internet-based intervention for alcohol abusers. Addiction, 104(12), 2023-32.

8. Cunningham, J. A., & Van Mierlo, T. Methodological issues in the evaluation of internet-based interventions for problem drinking. Drug and Alcohol Review, 28(1), 12-17.

9. Cunningham, J. A., Humphreys, K., Koski-Jännes, A., & Cordingley, J. Internet and paper self-help materials for problem drinking: Is there an additive effect? Addictive Behaviors, 30(8), 1517-1523.

10. Doumas DM & Hannah E. (2008). Preventing high-risk drinking in youth in the workplace: a web-based normative feedback program. Journal of Substance Abuse Treatment, 34(3), 263-71.

11. Doumas DM, McKinley LL & Book P. (2009). Evaluation of two Web-based alcohol interventions for mandated college students. Journal of Substance Abuse Treatment, 36(1), 65-74.

12. Doumas, DM; Haustveit, T Reducing heavy drinking in intercollegiate athletes: Evaluation of a Web-based personalized feedback program. SPORT PSYCHOLOGIST, 22 (2): 212-228 JUN 2008

13. Hallett, J., Maycock, B., Kypri, K., Howat, P., & McManus, A. Development of a web-based alcohol intervention for university students: Processes and challenges. Drug and Alcohol Review, 28(1), 31-39.

14. Hester, RK; Squires, DD. Web-based norms for the Drinker Inventory of Consequences from the Drinker's Checkup. JOURNAL OF SUBSTANCE ABUSE TREATMENT, 35 (3): 322-327 OCT 2008

15. Koski-Jannes, A; Cunningham, J; Tolonen, K Self-Assessment of Drinking on the Internet--3-, 6-and 12-Month Follow-Ups. ALCOHOL AND ALCOHOLISM, 44 (3): 301-305 MAY-JUN 2009.

16. Koski-Jannes, A; Cunningham, JA; Tolonen, K; et al. Internet-based self-assessment of drinking - 3-month follow-up data. Addictive Behaviors, 32(3), 533-542.

17. Kypri K, Hallett J, Howat P, McManus A, Maycock B, Bowe S, et al. (2009). Randomized controlled trial of proactive web-based alcohol screening and brief intervention for university students. Archives of Internal Medicine, 169(16), 1508-14.

18. Kypri K, Langley JD, Saunders JB & Cashell-Smith ML. (2007). Assessment may conceal therapeutic benefit: findings from a randomized controlled trial for hazardous drinking. Addiction, 102(1), 62-70.

19. Kypri K, Langley JD, Saunders JB, Cashell-Smith ML & Herbison P. (2008). Randomized controlled trial of web-based alcohol screening and brief intervention in primary care. Archives of Internal Medicine, 168(5), 530-6.

20. Kypri K, Saunders JB, Williams SM, McGee RO, Langley JD, Cashell-Smith ML, et al. (2004). Web-based screening and brief intervention for hazardous drinking: a double-blind randomized controlled trial. Addiction, 99(11), 1410-7.

21. Kypri, K. (2007). Methodological Issues in Alcohol Screening and Brief Intervention Research Methodology, trial, screening, brief intervention, alcohol, drinking. Substance Abuse, 28(3), 31. doi:10.1300/J465v28n03□04

22. Linke, S; McCambridge, J; Khadjesari, Z; et al. Development of a Psychologically Enhanced Interactive Online Intervention for Hazardous Drinking. ALCOHOL AND ALCOHOLISM, 43 (6): 669-674 NOV-DEC 2008

23. Murray E, Khadjesari Z, White IR, Kalaitzaki E, Godfrey C, McCambridge J, et al. (2009). Methodological challenges in online trials. Journal of Medical Internet Research, 11(2), e9.

24. Murray E, McCambridge J, Khadjesari Z, White IR, Thompson SG, Godfrey C, et al. (2007). The DYD-RCT protocol: an on-line randomised controlled trial of an interactive computer-based intervention compared with a standard information website to reduce alcohol consumption among hazardous drinkers. BMC Public Health, 7, 306.

25. Neighbors C, Lee CM, Lewis MA, Fossos N & Walter T. (2009). Internet-based personalized feedback to reduce 21st-birthday drinking: a randomized controlled trial of an event-specific prevention intervention. Journal of Consulting & Clinical Psychology, 77(1), 51-63.

26. Saitz R, Palfai TP, Freedner N, Winter MR, Macdonald A, Lu J, et al. (2007). Screening and brief intervention online for college students: the ihealth study. Alcohol & Alcoholism, 42(1), 28-36.

27. Schaus JF, Sole ML, McCoy TP, Mullett N & O'Brien MC. (2009). Alcohol screening and brief intervention in a college student health center: a

randomized controlled trial. Journal of Studies on Alcohol & Drugs. Supplement, (16), 131-41.

28. Walters ST, Vader AM & Harris TR. (2007). A controlled trial of web-based feedback for heavy drinking college students. Prevention Science, 8(1), 83-8.
29. Walters, S. T., Vader, A. M., Harris, T. R., Field, C. A., & Jouriles, E. N. Dismantling motivational interviewing and feedback for college drinkers: A randomized clinical trial. Journal of Consulting and Clinical Psychology, 77(1), 64-73.

30. Williams, J; Herman-Stahl, M; Calvin, SL; et al. Mediating mechanisms of a military web-based alcohol intervention. Drug and Alcohol Dependence, 100(3), 248-257.

FACE-TO-FACE COMPONENTS OR INTERACTION WITH PARTICIPANTS (N=2)

1. Finfgeld-Connett D. (2009). Web-based treatment for rural women with alcohol problems: preliminary findings. CIN: Computers, Informatics, Nursing, 27(6), 345-53.

2. Newton NC, Andrews G, Teesson M & Vogl LE. (2009). Delivering prevention for alcohol and cannabis using the Internet: a cluster randomised controlled trial. Preventive Medicine, 48(6), 579-84.

NON-RCT (N=2)

1. Linke, S; Murray, E; Butler, C; et al.Internet-based interactive health for intervention for the promotion of sensible drinking: Patterns of use and potential impact on members of the general public. JOURNAL OF MEDICAL INTERNET RESEARCH, 9 (2): Art. No. e10 2007

2. Riper H, Kramer J, Conijn B, Smit F, Schippers G & Cuijpers P. (2009). Translating effective web-based self-help for problem drinking into the real world. Alcoholism: Clinical & Experimental Research, 33(8), 1401-8.

REVIEW (N=1)

1. Walters, ST; Miller, E; Chiauzzi, E Wired for wellness: e-interventions for addressing college drinking JOURNAL OF SUBSTANCE ABUSE TREATMENT, 29 (2): 139-145 SEP 2005

PROTOCOL (N=1)

1. Wallace P, Linke S, Murray E, McCambridge J & Thompson S. (2006). A randomized controlled trial of an interactive Web-based intervention for reducing alcohol consumption. Journal of Telemedicine & Telecare, 12 Suppl 1, 52-4.

SUMMARY OF PROCEEDINGS (N=1)

1. Walters ST, Hester RK, Chiauzzi E & Miller E. (2005). Demon rum: high-tech solutions to an age-old problem. Alcoholism: Clinical & Experimental Research, 29(2), 270-7.

NO MAJOR WEB COMPONENT (N=1)

1. Turrisi R, Larimer ME, Mallett KA, Kilmer JR, Ray AE, Mastroleo NR, et al. (2009). A randomized clinical trial evaluating a combined alcohol intervention for high-risk college students. Journal of Studies on Alcohol & Drugs, 70(4), 555-67.

SECONDARY ANALYSIS (N=1)

1. Riper H, Kramer J, Keuken M, Smit F, Schippers G & Cuijpers P. (2008). Predicting successful treatment outcome of web-based self-help for problem drinkers: secondary analysis from a randomized controlled trial. Journal of Medical Internet Research, 10(4), e46.
