# Supplementary material for: Persuasive Features in Web-Based Alcohol and Smoking Interventions: A Systematic Review of the Literature
Source: J Med Internet Res. 2011 Jul 22;13(3):e46. doi: 10.2196/jmir.1559 (PMC3222186; doi:10.2196/jmir.1559)
Supplement: Supplementary file 2 [file jmir_v13i3e46_app2.pdf]

Tuomas Lehto and Harri Oinas-Kukkonen:
Persuasive Features in Web-Based Alcohol and Smoking Interventions – A Systematic Review of the Literature

MULTIMEDIA APPENDIX 2. EXCLUDED ARTICLES / SMOKING (N=14)

SUMMARY OF REASONS (N):

- Secondary analysis (4)
- Face-to-face components or interaction with participant (3)
- No major web component (3)
- Exploratory (2)
- Protocol (1)
- Non-RCT (1)

SECONDARY ANALYSIS (N=4)

1. Danaher BG, Smolkowski K, Seeley JR & Severson HH. (2008). Mediators of a successful web-based smokeless tobacco cessation program. Addiction, 103(10), 1706-12.
2. Danaher BG, Lichtenstein E, McKay HG & Seeley JR. (2009). Use of non-assigned smoking cessation programs among participants of a Web-based randomized controlled trial. Journal of Medical Internet Research, 11(2), e26.
3. Strecher VJ, Shiffman S & West R. (2006). Moderators and mediators of a web-based computer-tailored smoking cessation program among nicotine patch users. Nicotine & Tobacco Research, 8 Suppl 1, S95-101.
4. Strecher VJ, McClure JB, Alexander GL, Chakraborty B, Nair VN, Konkel JM, Greene SM, Collins LM, Carlier CC, Wiese CJ, Little RJ, Pomerleau CS, Pomerleau OF Web-based smoking-cessation programs: results of a randomized trial. American journal of preventive medicine. 34(5):373-81, 2008 May

FACE-TO-FACE COMPONENTS OR INTERACTION WITH PARTICIPANT (N=3)

1. Chen HH & Yeh ML. (2006). Developing and evaluating a smoking cessation program combined with an Internet-assisted instruction program for adolescents with smoking. Patient Education & Counseling, 61(3), 411-8.
2. Norman CD, Maley O, Li X & Skinner HA. (2008). Using the internet to assist smoking prevention and cessation in schools: a randomized, controlled trial. Health Psychology, 27(6), 799-810.
3. Mermelstein R, Turner L Web-based support as an adjunct to group-based smoking cessation for adolescents. Nicotine & tobacco research : official journal of the Society for Research on Nicotine and Tobacco. Vol.8 Suppl 1, pp.S69-76, 2006 Dec.

NO MAJOR WEB-COMPONENT (N=3)

1. Etter JF. (2009). Comparing computer-tailored, internet-based smoking cessation counseling reports with generic, untailored reports: a randomized trial. Journal of Health Communication, 14(7), 646-57.
2. Schumann, A., John, U., Baumeister, S., Ulbricht, S., Rumpf, H. & Meyer, C. (2008). Computer-tailored smoking cessation intervention in a general population setting in germany: Outcome of a randomized controlled trial. Nicotine and Tobacco Research, 10(2), 371-379.
3. Stoops WW, Dallery J, Fields NM, Nuzzo PA, Schoenberg NE, Martin CA, et al. (2009). An internet-based abstinence reinforcement smoking cessation intervention in rural smokers. Drug & Alcohol Dependence, 105(1-2), 56-62.

EXPLORATORY (FIVE DIFFERENT SITES) ->> INSUFFICIENT INFORMATION ABOUT THE SITES (N=2)

1. Pike, K. J., Rabius, V., McAlister, A., & Geiger, A. (2007). American cancer society's quitlink: Randomized trial of internet assistance. Nicotine and Tobacco Research, 9(3), 415-420.
2. Rabius V, Pike KJ, Wiatrek D & McAlister AL. (2008). Comparing internet assistance for smoking cessation: 13-month follow-up of a six-arm randomized controlled trial. Journal of Medical Internet Research, 10(5), e45.

PROTOCOL (N=1)

1. Kramer JJ, Willemsen MC, Conijn B, van Emst AJ, Brunsting S, Riper H Effectiveness of a web-based self-help smoking cessation intervention: protocol of a randomised controlled trial. BMC public health. Vol.9, pp.32, 2009.

NON-RCT (N=1)

1. Shegog R, McAlister AL, Hu S, Ford KC, Meshack AF & Peters RJ. (2005). Use of interactive health communication to affect smoking intentions in middle school students: a pilot test of the "Headbutt" risk assessment program. American Journal of Health Promotion, 19(5), 334-8.
